# Crystallization of Lanthanide—Ho^3+^ and Tm^3+^ Ions Doped Tellurite Glasses

**DOI:** 10.3390/ma15072662

**Published:** 2022-04-05

**Authors:** Julian Plewa, Małgorzata Płońska, Katarzyna Osińska, Robert Tomala

**Affiliations:** 1 Faculty of Science and Technology, Institute of Materials Engineering, University of Silesia in Katowice, 75 Pułku Piechoty Str. 1a, 41-500 Chorzów, Poland; katarzyna.osinska@us.edu.pl; 2 Institute of Low Temperature and Structure Research Polish Academy of Sciences, Okólna 2, 50-422 Wrocław, Poland; r.tomala@intibs.pl

**Keywords:** tellurite glasses, lanthanide dopants, crystallization

## Abstract

In the presented work, the tellurite glasses TeO_2_-WO_3_-ZnO doped with Tm^3+^ and Ho^3+^ ions were prepared by the same glass forming method. X-ray diffraction (XRD) and differential thermal analysis (DTA) techniques were used to study the effects of the forming technology on the thermal and structural properties of the fabricated glasses. After controlled crystallization of investigated glasses, the emission in the VIS- and NIR range was determined. The effect of silver doping on emission intensity was investigated. The value of the activation energy of the glass crystallization process was determined, while the E_a_ value for pure TeO_2_ glass was much lower than for tellurite glasses TeO_2_-WO_3_-ZnO.

## 1. Introduction

TeO_2_ host materials have a matrix that exhibits some intriguing properties. They have a wide transparency range (400–5000 nm [1,2,3]), high refractive index (>2 [4]), low photon energy (800 cm^−1^ [1,3]), and relatively low melting points (<800 °C [5]). Tellurium oxide TeO_2_ as a glass-forming material (Zachariasen’s GFA criterion Ed(TeO_2_) = 1136 kJ/mol [6]) together with numerous network modifiers such as WO_3_, ZnO, CdO, K_2_O, PbO, BaO, and Bi_2_O_3_ forms stable glasses in a wide range of concentrations [7].

Other properties characteristic of tellurite glasses observed through thermal analysis include a relatively high glass transition temperature T_g_ (>360 °C without alkali metal oxides, [3,8,9]) and a relaxation effect (the glass transition exhibits enthalpy relaxation [10,11,12]). This effect, also called “shadow glass transition”, occurs immediately after the glass transition temperature T_g_ is reached. Tellurite glasses also show remarkable absorption properties of gamma radiation, which was demonstrated in the work of [13].

Tellurite glasses are of great interest, especially when doped with rare-earth ions (Er, Eu, Tm, Ho) to obtain materials for infrared laser applications (2–3 µm) and optical devices in telecommunication [1,2,3,4,14,15,16]. Photonic applications of doped tellurite glasses may become even more interesting when doped with noble metals (Au and Ag nanoparticles), significantly increasing the effect of radiation emission [1,4,13,14,15,16]. This effect (functionalization of glasses with silver or gold) is attributed to the phenomenon of localized surface plasmon resonance (LPSR) and occurs for small Ag and Au nanoparticles [16].

There is a very extensive literature on tellurite glasses doped with Er^3+^ ions [17], Ho^3+^, and/or Tm^3+^ ions [7,18,19]. Such glasses are suitable for IR (mid-IR) phosphors when excited with 800 nm radiation [1,9,20,21]. Chen et al. [18] have confirmed the enhancement of 2 µm Ho^3+^ ion emission by Tm^3+^→ Ho^3+^ energy transfer. Later, Chen et al. [7] also proposed the use of these glasses for amplifiers for 1.47 µm radiation, especially since Ho^3+^ ions have been found to be a good co-dope for Tm^3+^ for this wavelength emission.

In addition to the above-mentioned functional properties of glasses [22,23], other important issues include their thermal stability as well as their crystallization ability, especially as these properties affect the photoresponse of glasses to excitation radiation.

It is a well-established fact that the thermal stability of glass depends on its composition as well as the concentration of its respective components. It is expressed by the difference in the characteristic temperatures T_x_ (onset of crystallization) and T_g_ (glass transition temperature) determined by thermal analysis: ΔT = T_x_ − T_g_. The higher the ΔT value, the more stable the glass. For tellurite glasses (not containing alkali metals), this value, according to literature data [2,4], is ΔT = 160–170 °C. Regarding the crystallization process of TeO_2_-WO_3_-ZnO tellurite glasses, the onset of crystallization temperature strongly depends on the concentration of WO_3_ and ZnO, and for pure TeO_2,_ it is about 350 °C [24], while for glasses with 30% WO_3_ content, it increases to about 480 °C [5].

The aim of the presented work is to verify these values by considering the Ho^3+^/Tm^3+^-doped and Ag-co-doped glasses. The focus is on comparing the crystallization kinetics of pure TeO_2_ glass, 70TeO_2–_20WO_3_-10ZnO glass doped with holmium and thulium (Ho^3+^/Tm^3+^), as well as that with holmium, thulium, and silver (Ho^3+^/Tm^3+^/Ag^+^). The manufactured glasses were also characterized optically.

The aim of the paper is to investigate in detail the thermal behavior and describe the kinetics and the luminescent properties of tellurite glasses.

## 2. Materials and Methods

### 2.1. Preparation of Samples

Tellurite glass with a composition of 70TeO_2_-20WO_3_-10ZnO and fixed concentrations of Ho^3+^ at 0.3% mol and Tm^3+^ at 0.5% mol (designation “glass”) was prepared by melt quenching technique and rapidly cooled on a steel plate. Glass melting conditions were as follows: 99.9% purity materials (Merck), Al_2_O_3_ crucible, temperature 850 °C, melting time 1 h. The glass was thermally annealed at 300 °C or 400 °C. All glass samples, namely made of pure TeO_2_ (designated “TeO_2_”) and doped as above with holmium and thulium but enriched with a silver (0.3% mol Ag+) in the form of AgCl (designated “glass + Ag”), were melted in the same fashion.

**Figure 1:**
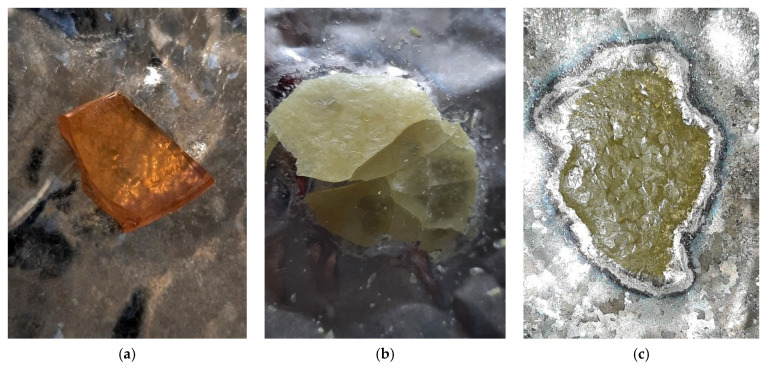
Glass form annealed at 400 °C (**a**) and solidified after annealing at 600 °C (**b**,**c**).

The size of the samples shown in Figure 1 was in the order of ca. 4 cm × 2 cm × 2–3 mm. From the macroscopic point of view, prepared samples were free from visible heterogeneities such as inclusions, cracks, or bubbles (i.e., Figure 1), so it was assumed that they were amorphous. It should also be added that while the glass cast and annealed at 300 °C had a yellowish color, it acquired an orange color when heated to 400 °C (Figure 1a). Moreover, the glass heated to 600 °C had a yellowish color again after cooling. As shown in Figure 1b,c, when the glasses had heated to in the range of 500–600 °C, they occurred in the liquid-plastic phase. In the temperature range mentioned above, a crystallization effect occurred in the glasses, which is characterized in more detail in the thermal analysis description of this work (Section 3.2).

### 2.2. Sample Examination

For the morphological study of the glass samples, scanning electron microscopy (SEM, HITACHI S-4700, Hitachi High-tech Group, Minato-ku, Tokyo, Japan) with a microanalysis system (EDXS- Thermo NORAN Vantage, Hitachi High-tech Group, Minato-ku, Tokyo, Japan) was used. The fractured surfaces of the glass were prearranged and coated with graphite.

A thermal analyzer (Derivatograph of Q-1500D- type Paulik–Paulik–Erdey system, the MOM-Hungary company, Budapest, Hungary) was used to analyze the crystallization process and to determine the characteristic temperatures T_g_ (glass transition), T_x_ (crystallization), T_p_ (exothermic peak), and T_f_ (melting). The activation energies of the crystallization process (E_a_) were determined from the crystallization temperature shift (T_p_) at different heating rates using Kissinger and Ozawa techniques [25].

X-ray phase analysis (with Empyrean XRD, Malvern Panalytical B.V., Almelo, the Netherlands) was used to determine the structural properties of the obtained glasses before and after heating at 400 °C. The data were collected in the 2θ range from 10° to 80°, in steps of 0.02 degrees, with an integration time of 4 s/step. Full pattern identification was made by using the X’Pert HighScorePlus software package (created by Malvern Panalytical B.V., Almelo, the Netherlands). Data from the PDF database (International Centre for Diffraction Data (ICDD^®^)) [26] were used as a reference for the structural analysis of glass material.

Luminescence studies were carried out with an Edinburgh FLS 980 spectrophotometer using an external excitation source–a CNI Laser 808 nm 2 W laser diode (Edinburgh Instruments, Livingston, UK). During the excitation with both the xenon lamp and the laser, the emissivity measurements were made for the range of 250–850 nm, while in the attached graphs, only the interesting wavelength ranges were shown.

## 3. Results

### 3.1. SEM Analysis

**Figure 2:**
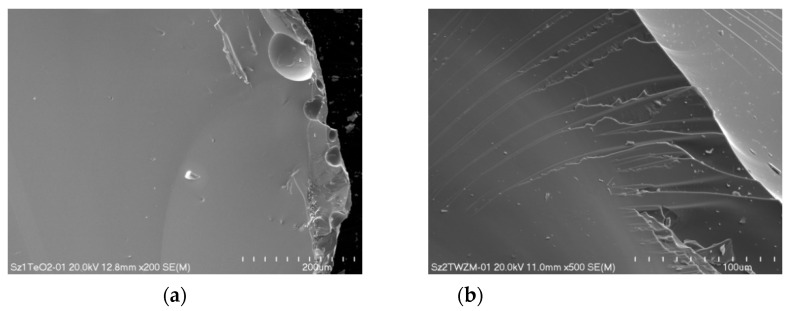
SEM picture of the morphology of the fractured surface of pure TeO_2_ glass (**a**) and doped glass (**b**).

The observation under a scanning electron microscope (SEM) confirmed the amorphous nature of the prepared glasses. Figure 2 shows typical examples of the morphology of the fractured surface of the investigated sample. In the microscale, the surface of the cast and stressed glasses was homogeneous and of high quality, in the case of pure TiO_2_ and a dopant material (Figure 2a,b). It should be noted that the non-annealed glasses showed a great tendency to crack and create scratches.

**Figure 3:**
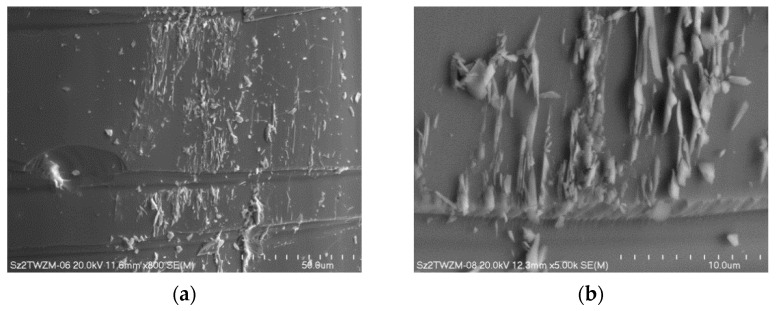
SEM image of the glass particles on the surface of dopant glasses occurred after rapid cooling, with magnification (**a**) 800× and (**b**) 5.0k×.

Since freshly cast dopant glass exhibits high internal stress, in some areas, a tendency to form elongated glass fragments collection on their surface was observed. Interestingly, the presence of glass particles on the surface occurred only after their rapid cooling. Example SEM images illustrating this tendency are shown in Figure 3.

**Figure 4:**
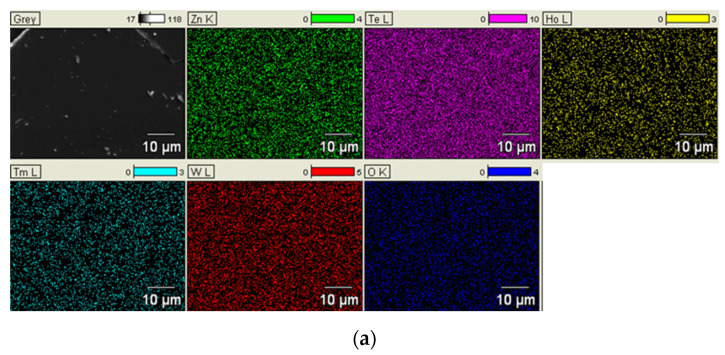

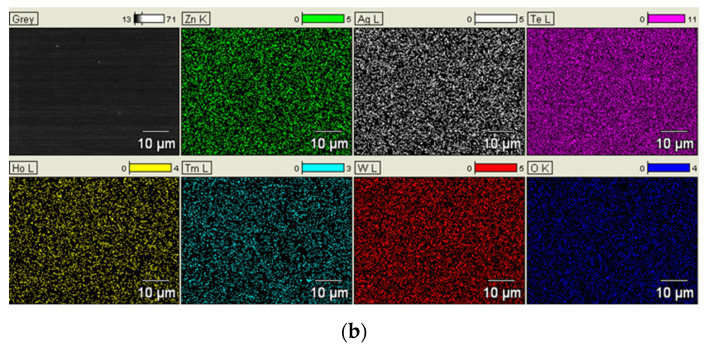
Results of EDXS mapping analysis of the “glass” (**a**) and “glass + Ag” (**b**).

The EDXS elementals mapping analyses of concentrations of Te, W, Zn, Ho, Tm, Ag, and O confirmed they were free from any contamination and indicated homogeneously distributed within the whole samples of dopant “glass” (Figure 4a) and “glass + Ag” (Figure 4b).

### 3.2. Thermal Analysis

Using a thermal analysis, DTA curves were determined for powdered glass samples, which were almost white in this form (chromatic diagram coordinates: x = 0.355, y = 0.366).

**Figure 5:**
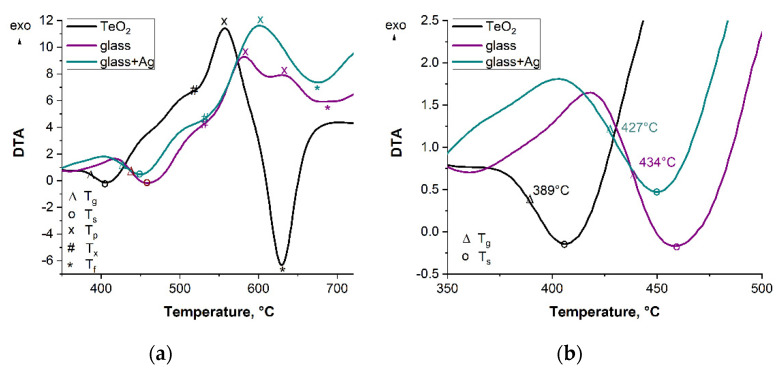
DTA curves for glasses: pure TeO_2_ and samples of “glass” and “glass + Ag” with marked characteristic temperatures (heating rate β = 10 °C/min). T_g_—temperature of glass transition; T_s_—temperature of glass relaxation; T_x_—onset of crystallization; T_p_—peak of crystallization; T_f_—temperature of melting (two ranges of temperatures, (**a**,**b**)).

DTA curves for three samples: the pure TeO_2_, the “glass,” and the “glass + Ag” are shown in Figure 5. The following characteristic temperatures were marked on the DTA curves: glass transition temperature T_g_ (the inflection point), relaxation temperature T_s_, crystallization onset temperature T_x_, crystallization peak temperature T_p_, and the melting peak temperature T_f_. The values of T_g_ and T_s_ for compound glasses are higher than for pure TeO_2_ glasses. The presented curves indicate two other aspects that need to be pointed out. All three tested glasses show a relaxation effect as well as a crystallization effect (exothermic effect with peak temperature T_p_). Glass from pure TeO_2_ exhibits a well-developed melting effect with a peak temperature of T_f_ (endothermic effect). For the “glass” sample, the melting effect (>650 °C) is fuzzy, and for silver doped glass (“glass + Ag”), it is entirely absent. Experimentally it was found that these two glasses already soften above 450 °C, whereas at 560 °C, they are already in a semi-liquid state.

Figure 5 shows typical thermograms of the tested glasses obtained through differential thermal analysis. The thermograms show an endothermic relaxation effect with a relaxation temperature T_s_; on the flank, there is the glass transition temperature T_g_.

As can be seen from Figure 5, an inflection point between 393 °C (for TeO_2_) and 429 °C (for “glass”) corresponding to the glass transition temperature (T_g_) can be observed for all samples, with a bigger value of T_g_ compared to pure TeO_2_, which was caused by the addition of WO_3_ and ZnO. From the obtained correlations, one can also deduce the stability of the glasses, which is defined as the difference between the glass transition temperature T_g_ and the temperature of the crystallization onset T_x_. The calculated values of ΔT = T_x_ − T_g_ indicate that the tested glasses have a relatively narrow stability range (>98 °C), slightly smaller than that of pure TeO_2_ glass (ΔT = 129 °C).

The DTA curves obtained for tellurite glasses include the following points of interest associated with the dynamic transformations occurring when the samples are heated, namely the glass transition temperature T_g_, the relaxation range with temperature T_s_, the crystallization range with crystallization onset temperature T_x_, the crystallization peak temperature T_p_, and the melting peak temperature T_f_. It should be noted that the crystallization process analyzed takes place in the regime of heating the samples and during the process of continuous reduction of the glass viscosity, and the appearance of a single peak corresponding to relaxation is a characteristic feature of the tested glasses.

### 3.3. Analysis of Kinetics of the Glass Crystallization, by the Kissinger Method

**Figure 6:**
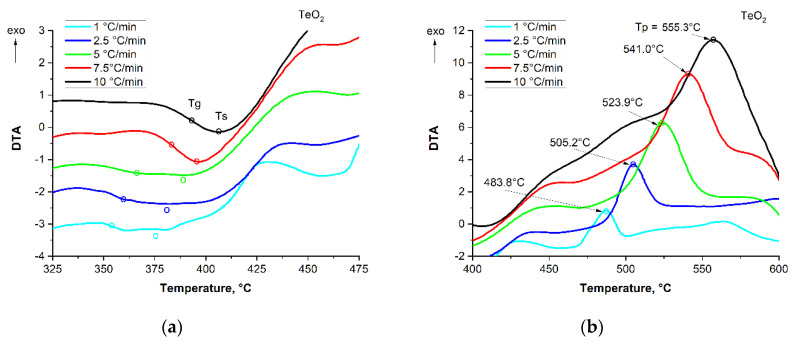

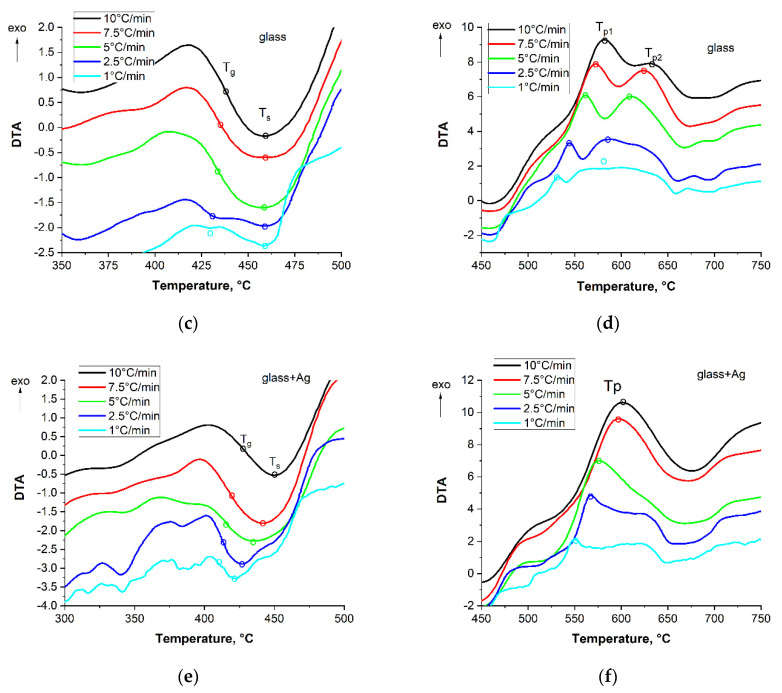
DTA curves for the crystallization of pure TeO_2_ glass (**a**,**b**), “glass” (**c**,**d**), and “glass + Ag” (**e**,**f**).

To study kinetic aspects of glass crystallization, the well-known Kissinger method [25]—an analytical procedure for measuring DTA at different heating rates—was used. Using the well-known analytical procedure of measuring DTA at different heating rates and determining the temperature of the exothermic peak maximum T_p_, the crystallization process was compared between the glasses and pure TeO_2_. Results were illustrated by typical curves, registered for measurements at the heating rate of β = 1, 2.5, 5, 7.5, and the step 10 °C/min, as shown in Figure 6. One can see that the obtained glass crystallization curves differ both in their shape and intensity. By introducing WO_3_ and ZnO into TeO_2_ glass, the crystallization process takes a qualitatively different course, with this ternary glass crystallizing in two stages in contrast to pure TeO_2_ glass. It can be added that the curves obtained representing the thermal properties of the heated glass samples do not, however, reflect the thermo-viscosity of the samples, which strongly decreases along with the increasing temperature.

Using the Kissinger procedure [25], it is possible to determine the kinetic parameter in the form of the apparent crystallization energy of the glasses. For this purpose, the exothermic peak temperature T_p_ is used. The values given for T_g_, T_s_, and T_p_ are dynamic characteristic values, as each of them moves to higher temperatures as the heating rate increases. The characteristic temperatures, including T_g_, T_s_, and the peak crystallization temperature T_p_ of all samples determined on the basis of the DTA curves, are summarized in Table 1.

**Figure 7:**
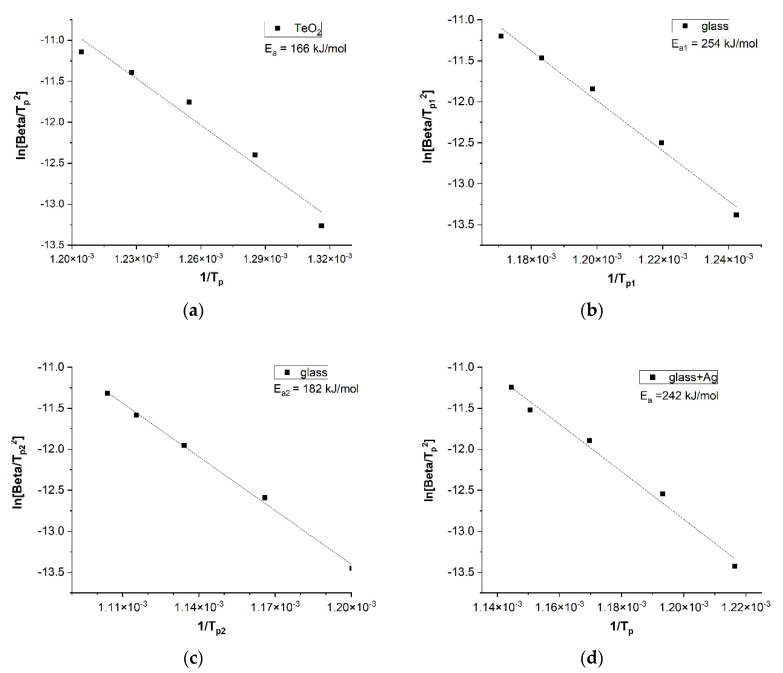
Kissinger relation for the crystallization process of TeO_2_ glass (E_a_ = 166 kJ/mol) (**a**), “glass” (E_a_1 = 256 kJ/mol, E_a_2 = 182 kJ/mol) (**b**,**c**), and “glass + Ag” (E_a_ = 242 kJ/mol) (**d**).

The energy barrier of the crystallization process is customarily determined in terms of the apparent activation energy. This parameter allows for quantifying the differences in the crystallization process for the different glasses under comparison. The relationships that stem from the kinetic equation according to the Kissinger method [25] are presented in Figure 7.

The relationships presented have two important aspects. Firstly, they deviate from Arrhenius behavior, and, secondly, the apparent activation energy for glasses made of pure TeO_2_ is much smaller than for TeO_2_, WO_3_, and ZnO glasses. This means that pure tellurium oxide glass crystallizes more easily and at lower temperatures. This can be related to the fact already pointed out in the literature that pure tellurium oxide glass is difficult to obtain [21]. The determined values of the apparent activation energy Ea for glasses composed of TeO_2_, WO_3_, and ZnO are higher than for glasses of pure TeO_2_.

Tellurite glass containing WO_3_ and ZnO (“glass”) shows remarkably different DTA curve behavior and crystallizes in two stages. This trend is also revealed for low heating rates in the “glass + Ag” samples.

### 3.4. XRD Analysis

**Figure 8:**
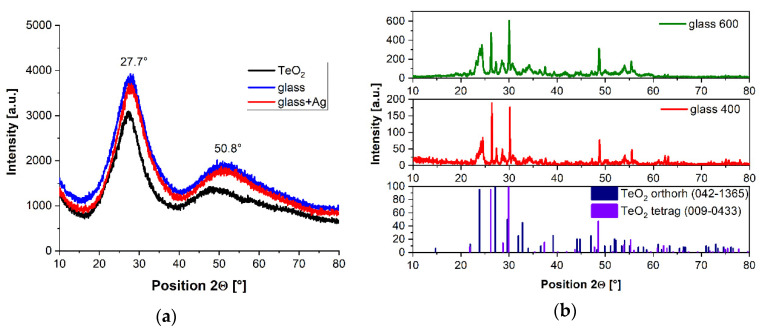
XRD results for glasses following their annealing at 300 °C (**a**) and glasses after additional heat treatment at 400 °C and at 600 °C (**b**).

The crystalline phases form when the glasses are heated, also indicated during the structural analyses. The obtained results have been verified by matching the tetragonal (ICDD^®^ 00-042-1365) patterns and orthorhombic (ICDD^®^ 00-009-0433) TeO_2_ standards. Figure 8 shows the results of XRD analysis for powdered glass samples previously heat-treated at three different temperatures: 300 °C, 400 °C, and 600 °C.

The XRD results for glasses annealed at a temperature of 300 °C (Figure 8a) do not indicate the presence of crystalline phases. They show, however, the courses characteristic for the amorphous oxide phase, with exhibits broad diffuse scattering in the range 2θ = 20–30°. It demonstrates the amorphous nature of glass and indicates the lack of long-range order in its atomic structure.

In Figure 8b, one can see that the glass, after being heat-treated at 400 °C and 600 °C, showed the presence of crystalline tellurium oxide. They were identified as two polymorphic types—tetragonal and orthorhombic phases of TeO_2_. Interestingly, at the temperature of ~600 °C, the glass samples were in the form of the liquid-plastic phase.

### 3.5. Optical Properties of the Glasses

**Figure 9:**
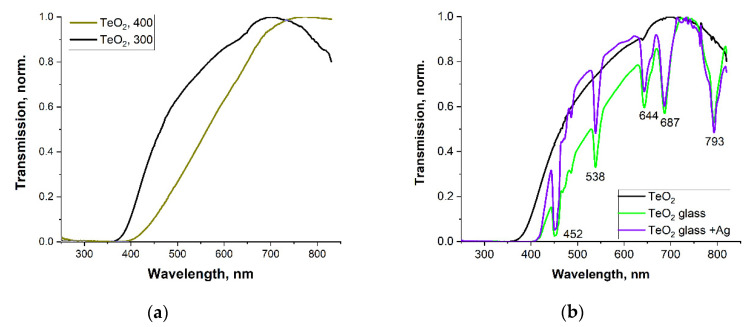
Transmission spectra for the pure TeO_2_ glass annealed at 300 °C and 400 °C (**a**) and the pure TeO_2_ glass, “glass”, and “glass + Ag” at 300 °C (normalized curves) (**b**).

The prepared glass samples are transparent and yellowish in color. The color of the samples is strongly dependent on the annealing temperature. For instance, when glasses were annealed at 400 °C, their color changed from yellowish to dark reddish (Figure 9a).

Tellurite glasses easily absorb large amounts of UV light, up to above 400 nm. Glasses doped with Tm and Ho exhibit absorption associated with transitions from the ground state to the excited levels of the following ions: 452 nm (Ho^3+^: ^5^F_3_), 537 nm (Ho^3+^: ^5^F_4_), 642 nm (Tm^3+^: ^1^G_4_), 686 nm (Tm^3+^: ^3^F_2_), and 794 nm (Tm^3+^: ^3^H_4_) (Figure 9b).

By annealing at 400 °C, the color of the glass changed from yellowish to reddish, making it impermeable to radiation of up to 500 nm.

Ho^3+^ and Tm^3+^ ions are known to exhibit luminescence in a wide range of the visible spectrum. When doped glasses are excited at different λ wavelengths, different emission responses can be obtained. Particularly energetically strong wavelengths in the UV range (λ_ex_ = 225 nm) give stronger emission than excitation with blue light (λ_ex_ = 450 nm). From the application point of view, the investigated glasses are preferably excited in the infrared range.

**Figure 10:**
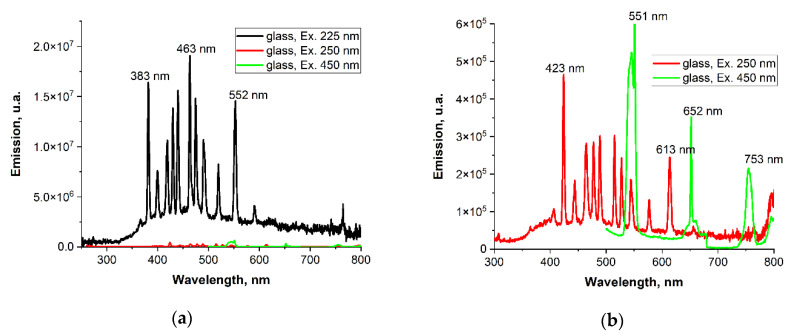
Excitation luminescence spectra for ‘glass’ monitoring emission at λ_ex_ = 225, 250, and 450 (**a**) as well as at 250 and 450 nm (**b**).

Figure 10 shows the excitation luminescence spectra monitoring emission at 547 and 800 nm, which correspond to the ^5^F_4_,^5^S_2_→^5^I_8_ transitions of the Ho^3+^ ion and the ^3^H_4_→^3^H_6_ transition of the Tm^3+^ ion, respectively. The excitation spectra confirm the energy transfer occurring between Ho^3+^ and Tm^3+^ ions, and thus, the band at 547 nm associated with the ^5^I_8_ → ^5^F_4_,^5^S_2_ transition of the Ho^3+^ ion effectively excites the emission of the Tm^3+^ ion monitored at 800 nm.

**Figure 11:**
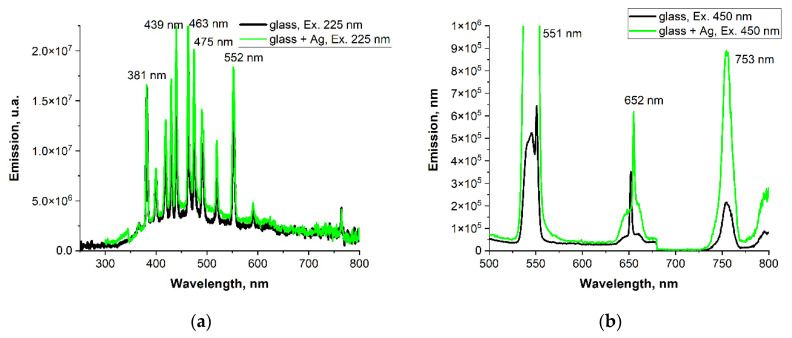
Comparison of emission spectra for doped glasses upon (**a**) 225 nm and (**b**) 450 nm excitation, with the addition of silver and without the addition of silver.

Figure 11 presents emission spectra of glasses with and without silver; 350 and 450 nm lines, corresponding to the absorption bands of Thulium and Holmium ions, have been used for excitation.

Emission lines of Ho^3+^: ^5^F_5_→^5^I_8_ (deep red—657 nm) and ^5^F_4_: ^5^S_2_→^5^I_8_ (green—547 nm) and the weaker line ^5^F_4_: ^5^S_2_ -→^5^I_7_ (red—754 nm)—upon 450 nm excitation can be seen, while the intense line upon 350 nm excitation is from Tm^3+^: ^3^H_4_→^3^H_6_.

It can be seen that silver doping leads to the drop in luminescence intensity of both holmium and thulium ions. In addition, the admixture of silver enhances the emission in the visible range.

**Figure 12:**
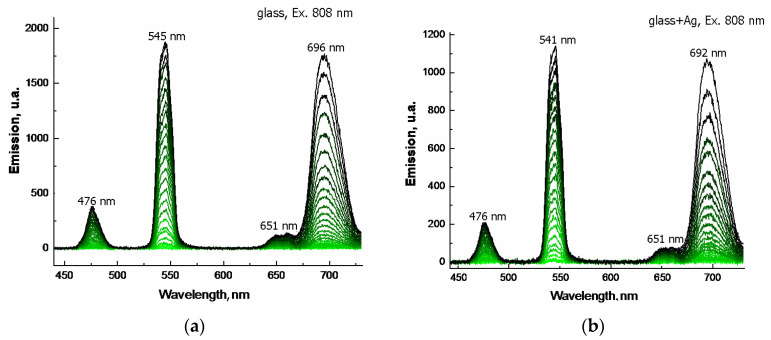
Emission spectra for doped glasses upon λ_ex_ = 808 nm diode excitation; glasses without (**a**) and with (**b**) silver, respectively.

Figure 12 shows the emission spectra for doped glasses when excited with IR radiation at 808 nm. Here, too, emission in the visible range is achieved, with three bands of radiation: 480 nm, 545 nm, and 695 nm. Although Ho^3+^ does not have an absorption band corresponding to a laser diode at ~800 nm, Tm^3+^ ions do absorb such radiation, and the excited levels of Ho^3+^ ions are occupied via energy transfer.

The presented emission spectra were obtained for glasses in reflected light. They indicate that silver doping, in this case, negatively affects the intensity of emission in reflected light. Due to the focusing of the excitation beam, the lower emission intensity of silver-doped glass is also most likely related to its higher temperature and, consequently, to thermal quenching [22]. The presented spectra indicate that up-conversion gives a luminescence intensity of blue emission (476 nm) much lower than that of green (545 nm) and red (654 nm) emissions.

## 4. Discussion

The studies described above involved the standard procedure of melting the glasses at 850 °C. After the melting process, the glass samples were cast onto a steel plate and then solidified and carefully transferred to a furnace, where each sample was annealed at four selected temperatures: 300, 400, 500, and 600 °C, while for DTA thermal measurements and optical characteristics, glass samples annealed at 300 °C were used. Our analysis has shown that the melting point of tellurite glasses was higher than the original melting conditions by other authors [3,13] because the glass did not contain liquid additives such as B_2_O_3_ or alkali metal oxides.

As mentioned, the research mainly focused on the crystallization of tellurite glasses, which occur in glasses during their heating. This process was analyzed based on DTA measurements because the crystallization of glasses with heating shows a measurable exothermic effect. It is also interesting to note that this crystallization process takes place during the solidification of the liquid glass and is visible in the appearance of the surface microstructure. In this case, the subject of SEM analysis was the microstructure of the glass surface. It was observed that the surface of the analyzed glasses was homogeneous and smooth, although it contained elongated, irregular glass particles in some parts. A similar collection of regular glass particles was described in the work of [13], but they were on tellurite glasses’ surface containing B_2_O_3_. The high quality of prepared materials was confirmed by the EDXS mapping analyses, which show the distribution of all components (Te, W, Zn, Ho, Tm, O, and Ag) homogeneously in samples.

Thermal analysis also shows that the tellurite glasses under investigation have a glass transition temperature T_g_ above ca. 430 °C and a crystallization onset about 100 °C higher. It should be added here that the glass samples tested become plastic (soft) above a temperature of about 450 °C so that the crystallization process (and the glass becoming opaque) takes place in a viscous plastic mass. Glass-making practice shows that there is a range of extrusion near the T_g_ temperature and a range of fiber drawing above the T_p_ temperature.

The thermal stability of the tested glasses, defined as ΔT = T_x_ − T_g_, was lower than 100 °C, which does not suggest their high stability highlighted in the literature [2,4,20,21,22,23,24,25,26,27,28,29,30].

The conducted studies have confirmed the presence of a relaxation effect for tellurite glasses, which is an endothermic effect on the DTA curve. It is assumed that relaxation relates to the cooperative movement of atoms, i.e., it is directly related to viscous flow [29].

This relatively high glass transition temperature T_g_ > 450 °C is helpful for the glass fiber design process. For the studied glasses from the TeO_2_-WO_3_-ZnO system, values of T_g_ > 427 °C and T_p_ > 580 °C and high values of activation energy for the crystallization process in the range of 180–250 kJ/mol were recorded. As a comparison, TeO_2_-WO_3_ glasses exhibit T_g_ > 327 °C and T_p_ > 500 °C, as well as E_a_ values in the range of 228–379 kJ/mol [5,31].

The presence of the double crystallization peak obtained in the “glass” samples has already been described for glasses containing TeO_2_ and WO_3_. Thus, two values of the apparent activation energy, E_a_1 and E_a_2, are calculated, with usually the first value being higher than the second [31,32]. In the study conducted, these values, related to the exothermic transformation for the “glass” sample, are E_a_1 = 254 kJ/mol and E_a_1 = 182 kJ/mol, respectively. However, in the case of the “glass + Ag” sample, a similar value was obtained, i.e., E_a_ = 242 kJ/mol.

The effect resulting from the double crystallization peak of the “glass” samples can be explained by a change in the mobility of the structural units of the glass—the polyhedral TeO_3_ and TeO_4_—with increasing temperature [24].

With regard to the glass samples made (in the corundum crucible) from pure TeO_2_, the T_g_ and T_p_ values obtained are lower than those for “glass” and “glass + Ag” samples. It can be added that glass from pure TeO_2_ was relatively easy to obtain, which contradicts the literature reports of difficulties in producing such glass, e.g., [5]. For glass crystallization from pure TeO_2_, a lower value of the apparent activation energy was obtained, which was E_a_ = 166 kJ/mol.

The tellurite glasses studied through doping with Tm^3+^ and Ho^3+^ ions exhibit interesting optical properties when excited in both reflected and transmitted light. According to the results presented, the transmission spectra of the doped glasses show numerous radiation absorption effects from Tm^3+^ ions (687 nm, 793 nm) and from Ho^3+^ ions (452 nm, 538 nm, and 644 nm). The studies made use of the well-known fact that lanthanide ions (Ln^3+^), including Tm^3+^ and Ho^3+^, have multiple energy levels (rich energy-level structure), which allows for efficient frequency conversion, including DC (down-conversion) and UC (up-conversion) through energy transfer.

For selected exciting radiation values λ_ex_ = 250 nm and 450 nm, a wide emission range was obtained. Emission lines were shown to originate from Ho^3+^: ^5^F_4_, ^5^S_2_→^5^I_7_ (deep red—657 nm) and ^5^F_4_, ^5^S_2_→^5^I_8_ (green—547 nm), ^5^F_4_,^5^S_2_→^5^I_4_ (red—754 nm) upon 450 nm excitation, while upon 350 nm excitation, emission of Tm^3+^ ion is observed in the NIR range: ^3^H_4_→^3^H_6_ (800 nm). This means that if tellurite glasses doped this way are excited with 450 nm radiation Ho^3+^, ion radiative transitions dominate, while Tm^3+^ ion radiative transitions dominate at about 350 nm. It should be added that tellurite glasses doped with Ho^3+^ and Tm^3+^ ions lie in the sphere of interest of infrared fluorescence spectroscopy, e.g., [3,7,33].

In the case of excitation of the “glass” with IR diode, emission in the visible VIS range was also achieved. The obtained result indicates that up-conversion occurs in tellurite glasses co-doped with Tm and Ho, i.e., upon excitation, around 808 nm emission of visible green light is observed, i.e., 480 nm and 540 nm, and red light at 695 nm. This way, a strong visible up-conversion emission was obtained due to the absorption of 808 nm radiation by Tm^3+^ ions acting as a sensitizer and energy transfer to Ho^3+^ ions (Tm^3+^ → Ho^3+^) acting as an activator. The spectrum of doped tellurite glass contains three emission peaks at 480 nm, 540 nm, and 695 nm, which are caused by the respective transitions: Tm^3+^: ^1^G_4_ → ^3^H_6_ and Ho^3+^: (^5^S_2_,^5^F_4_)→ ^5^I_8_, and Tm^3+^: ^3^F_2,3_ → ^3^H_6_. The 480 nm blue emission is relatively weak, while the reverse energy transfer from Tm^3+^ to Ho^3+^ gives the two remaining intense colors—green and red. This has confirmed the effects previously reported in Li et al. [3], demonstrating green light emission for glass tellurite fibers doped with Tm and Ho and excited with an 800 nm diode.

The novelty of the presented results is that the obtained characteristic values of Tg ∆T glasses differ from those given in the literature and, in particular, indicate that the stability of the tested glasses is lower than the values given in the literature [2]. Apart from that, paying attention to the fact that the crystallization process of the glasses (visualized by the exothermic peak) takes place not in the solid phase but in the plastic-liquid phase should open up a new point of view on this phenomenon.

We also confirmed an essential property of Tm and Ho doped glasses, namely obtaining green light radiation due to the up-conversion effect.

## 5. Conclusions

In the presented study on tellurite glasses composed of TeO_2_-WO_3_-ZnO doped with Ho^3+^ and Tm^3+^ ions, glass transition T_g_ values above 430 °C were obtained. It was found that such glasses crystallize in the liquid-plastic state above 530 °C. XRD analysis confirmed that tellurium oxide crystallizes from the amorphous phase of the glass. The determined activation energy of the crystallization process of TeO_2_-WO_3_-ZnO glass lies in the range above 250 kJ/mol and is much higher than the activation energy of crystallization of pure amorphous phase TeO_2_.

In conclusion, due to doping with holmium Ho^3+^ and thulium Tm^3+^ ions, the studied tellurite glasses are very interesting functional optical materials and can exhibit emissivity in a wide range of wavelengths. Depending on the excitation wavelength, emissivity can be obtained in the visible range, both by excitation with UV and blue light as well as infrared radiation. The luminescence spectra show emission bands specific for the electron transition from the excited states to the ground states of the dopant ions. The presence of silver dopant in the investigated tellurite glasses enhances the luminescence with blue light excitation, which did not occur with infrared radiation excitation.

## Figures and Tables

**Table 1:** Characteristic temperatures of glass crystallization as a function of beta (β) heating rate.

β [°C/Min]	1 °C/Min	2.5 °C/Min	5 °C/Min	7.5 °C/Min	10 °C/Min
T_g_ (TeO_2_)	350.3	355.0	376.1	382.7	393.1
T_s_ (TeO_2_)	374.8	381.5	388.2	395.7	406.1
T_p_ (TeO_2_)	483.1	505.2	523.9	541.0	555.3
T_g_(glass)	429.0	431.1	433.9	435.0	437.1
T_s_ (glass)	459.4	459.0	458.7	458.3	459.0
T_p1_ (glass)	531.8	546.9	561.3	572.2	581.1
T_p2_ (glass)	560.1	584.7	608.7	623.6	632.8
T_g_ (glass + Ag)	402.3	411.5	414.8	419.1	427.1
T_s_ (glass + Ag)	420.7	426.3	434.3	441.5	450.4
T_p_ (glass + Ag)	549.1	565.1	581.9	596.2	600.7

## Data Availability

All data have contained within the article.
